# A population-based cohort study of perinatal mental illness following traumatic brain injury

**DOI:** 10.1017/S2045796025000150

**Published:** 2025-03-13

**Authors:** Hilary K. Brown, Kinwah Fung, Andrea Mataruga, Rachel Strauss, Vincy Chan, Natalie Urbach, Tatyana Mollayeva, Angela Colantonio, Eyal Cohen, Cindy-Lee Dennis, Joel G. Ray, Natasha R. Saunders, Simone N. Vigod

**Affiliations:** 1 Department of Health & Society, University of Toronto Scarborough, Toronto, ON, Canada; 2 Dalla Lana School of Public Health, University of Toronto, Toronto, ON, Canada; 3 Women’s College Research Institute, Women’s College Hospital, Toronto, ON, Canada; 4 ICES, Toronto, ON, Canada; 5 KITE Research Institute-Toronto Rehabilitation Institute, University Health Network, Toronto, ON, Canada; 6 Rehabilitation Sciences Institute, Temerty Faculty of Medicine, University of Toronto, Toronto, ON, Canada; 7 School of Medicine, Queen’s University, Kingston, ON, Canada; 8 Institute of Health Policy, Management and Evaluation, University of Toronto, Toronto, ON, Canada; 9 Department of Occupational Science and Occupational Therapy, Temerty Faculty of Medicine, University of Toronto, Toronto, ON, Canada; 10 Hospital for Sick Children, Toronto, ON, Canada; 11 Edwin SH Leong Centre for Healthy Children, University of Toronto, Toronto, ON, Canada; 12 Department of Pediatrics, Temerty Faculty of Medicine, University of Toronto, Toronto, ON, Canada; 13 Lawrence Bloomberg Faculty of Nursing, University of Toronto, Toronto, ON, Canada; 14 Lunenfeld-Tannenbaum Research Institute, Mount Sinai Hospital, Toronto, ON, Canada; 15 Li Ka Shing Knowledge Institute, Unity Health Toronto, Toronto, ON Canada; 16 Department of Psychiatry, Temerty Faculty of Medicine, University of Toronto, Toronto, ON, Canada

**Keywords:** brain injuries, cohort study, mental disorders, perinatal care, traumatic brain injury

## Abstract

**Aims:**

To examine the risk of perinatal mental illness, including new diagnoses and recurrent use of mental healthcare, comparing women with and without traumatic brain injury (TBI), and to identify injury-related factors associated with these outcomes among women with TBI.

**Methods:**

We conducted a population-based cohort study in Ontario, Canada, of all obstetrical deliveries to women in 2012–2021, excluding those with mental healthcare use in the year before conception. The cohort was stratified into women with no remote mental illness history (to identify new mental illness diagnoses between conception and 365 days postpartum) and those with a remote mental illness history (to identify recurrent illnesses). Modified Poisson regression generated adjusted relative risks (aRRs) (1) comparing women with and without TBI and (2) according to injury-related variables (i.e., number, severity, timing, mechanism and intent) among women with TBI.

**Results:**

There were *n* = 12,724 women with a history of TBI (mean age: 27.6 years [SD, 5.5]) and *n* = 786,317 without a history of TBI (mean age: 30.6 years [SD, 5.0]). Women with TBI were at elevated risk of a new mental illness diagnosis in the perinatal period compared to women without TBI (18.5% vs. 12.7%; aRR: 1.31, 95% confidence interval [CI]: 1.24–1.39), including mood and anxiety disorders. Women with a TBI were also at elevated risk for recurrent use of mental healthcare perinatally (35.5% vs. 27.8%; aRR: 1.18, 95% CI: 1.14–1.22), including mood and anxiety, psychotic, substance use and other mental health disorders. Among women with a history of TBI, the number of TBI-related healthcare encounters was positively associated with an elevated risk of new-onset mental illness.

**Conclusions:**

These findings demonstrate the need for providers to be attentive to the risk for perinatal mental illness in women with a TBI. This population may benefit from screening and tailored mental health supports and treatment options.

## Introduction

Perinatal mental illness is a common and costly complication of childbearing, affecting one in five women and other birthing people in pregnancy and postpartum (O’Hara and Wisner, 2014). Perinatal mental illnesses, including widespread disorders such as depression and anxiety and rare but serious illnesses such as bipolar and psychotic disorders, can have major negative impacts on long-term maternal and child wellbeing (Kingston and Tough, 2014; Meltzer-Brody and Stuebe, 2014). Traumatic brain injury (TBI) is an injury to the brain caused by an external force and can range from mild to severe (World Health Organization, 2006). One-third of people with TBI are female (Thurman *et al.*, 1999). There are nearly 400 emergency department visits and 50 hospitalizations per 100,000 females per year in Canada (Public Health Agency of Canada, 2020), with intimate partner violence being an important contributor(Haag *et al.*, 2022). Outside of pregnancy, women with TBI are at higher risk for mental illness than women without TBI as well as men with TBI (Chan *et al.*, 2022a; Farace and Alves, 2000; Perry *et al.*, 2016; Yeh *et al.*, 2020). Yet, little is known about the risk for perinatal mental illness in this population.

Women with TBI may be differentially susceptible to mental illness perinatally. The social (e.g., violence; Langlois *et al.*, 2006) and health disparities (e.g., neurological comorbidities; Chan *et al.*, 2017; Haag *et al.*, 2016) experienced by women with TBI are risk factors for perinatal mental illness (Brown *et al.*, 2019; Yang *et al.*, 2022). The specific sequelae of TBI may also contribute to an elevated risk. People with TBI often report ongoing physical, cognitive, social and emotional symptoms, such as headaches, irritability, sleep issues, executive function limitations and problem-solving difficulties (Kieffer-Kristensen and Teasdale, 2011; Mollayeva *et al.*, 2016; Pryor, 2004). Such issues may be exacerbated in the perinatal period, especially postnatally when women experience sleep deprivation and childcare stressors (e.g., infant crying; Pryor, 2004). Perinatal hormonal fluctuations could also impact the vulnerable brain (Brunton and Russell, 2010). In a study of 208 women, Colantonio *et al.* (2010) found 50% of women with a history of TBI reported ‘postpartum problems’ vs. 24% of those without TBI. The third most common problem was depression, reported by 19% of women with TBI. To date, however, there is no population-level evidence on this topic; such evidence is essential for strategizing health service provisions. Therefore, in a population-based cohort study, we compared the risks of new mental illness diagnoses and recurrent use of mental healthcare from conception to 365 days post-delivery in women with vs. without TBI. We also examined injury-related factors associated with these outcomes among women with a TBI.

## Method

### Study design and setting

We undertook a population-based cohort study in Ontario, Canada, using data from ICES (Toronto), an independent, non-profit organization that holds complete, reliable administrative health data derived from the healthcare encounters of Ontario’s 14.7 million residents (Williams and Young, 1996). We accessed and analysed the following datasets at ICES, linked at the individual level using a unique encoded identifier: the Canadian Institute for Health Information Discharge Abstract Database, including the derived MOMBABY dataset; the Ontario Mental Health Reporting System; the National Ambulatory Care Reporting System; the Ontario Health Insurance Plan dataset; the Registered Persons Database; the Immigration, Refugees and Citizenship Canada Permanent Resident Database; and the Census (Table S1). Use of data for this project was authorized under section 45 of Ontario’s *Personal Health Information Protection Act* (PHIPA), which does not require ethics board review. We followed the Strengthening the Reporting of Observational Studies in Epidemiology (STROBE) guidelines in our reporting (von Elm *et al.*, 2008).

### Study population

**Figure 1.**
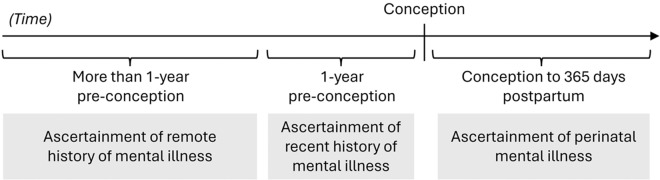
Illustration of the cohort design. We excluded women who used mental health services in the 1-year pre-conception because they were considered to be in active treatment for mental illness. We then stratified our cohort by remote history of mental illness status, defined based on ≥2 physician visits within 2 years of each other, or ≥1 emergency department visits or hospital admissions with a mental illness diagnosis between database inception and 1-year pre-conception. This allowed us to ascertain new mental illness diagnoses (in those without a remote history of mental illness) and recurrent use of mental healthcare (in those with a remote history of mental illness) perinatally.

We identified 15- to 49-year-old females (based on sex recorded on provincial health insurance cards) with an obstetrical delivery (i.e., livebirth or stillbirth after 20 weeks gestation) conceived between 1 April 2012 and 31 March 2021. Conception dates were estimated by subtracting gestational age at birth, most often ascertained using first trimester ultrasound, from the delivery date (You *et al.*, 2008). We excluded those who used mental health services in the year before conception because they were considered to be in active treatment for mental illness (Figure S1). One of the strongest risk factors for perinatal mental illness is history of mental illness (O’Hara and Wisner, 2014), and there is a bi-directional relationship between TBI and mental illness in the general population (Chan *et al.*, 2022a; Liao *et al.*, 2012; McHugo *et al.*, 2017; Yeh *et al.*, 2020). Thus, we stratified our cohort by remote history of mental illness, defined based on ≥2 physician visits within 2 years of each other, or ≥1 emergency department visits or hospital admissions with a mental illness diagnosis between database inception and 1-year pre-conception. This allowed us to ascertain new mental illness diagnoses and recurrent use of mental healthcare perinatally (Figure 1).

### Measures

We used a validated Centers for Disease Control and Prevention algorithm (Hedegaard *et al.*, 2016; Table S2) to identify women with a TBI recorded in the ‘most responsible diagnosis’ field in ≥1 emergency department visits or hospitalizations in the 10 years before conception. This algorithm has a positive predictive value of 96.9% relative to clinician-documented TBI (Warwick *et al.*, 2020). We also (1) examined TBI recorded in any diagnostic field (which better captures individuals with polytrauma but also captures those with a prior TBI unrelated to the encounter; Public Health Agency of Canada, 2020) and (2) used a sensitive definition of TBI (which includes open wounds of the head, fracture of the orbital floor and crushing injuries of the face and head – which often accompany, but do not necessarily indicate, TBI) (Brown *et al.*, 2024). Those without TBI in the 10 years before conception were the referent.

In those with a TBI recorded in the ‘most responsible diagnosis’ field in the 10 years before conception, we additionally measured injury-related variables: (1) the number of acute healthcare encounters for TBI (separated by ≥24 hours); (2) severity of the most severe TBI, based on a combination of hospital admission and discharge destination (i.e., emergency department visit only, or hospital admission and discharged home or to another facility; Brown *et al.*, 2024); and (3) time since the most recent acute healthcare encounter for TBI. For the TBI most proximal to conception, we also ascertained the (4) mechanism of injury (i.e., motor vehicle collision, struck by/against, falls, other; Hedegaard *et al.*, 2016) and (5) intent (i.e., intentional, unintentional/undetermined/other; Centers for Disease Control and Prevention, 2015).

Perinatal mental illness was defined as a healthcare encounter for mental illness between conception and 365 days postpartum, captured in ≥1 visits to a primary care provider, paediatrician, obstetrician or psychiatrist; emergency department visits; or hospital admissions with a mental illness diagnosis (Table S3). Perinatal mental illness was then further categorized by (a) timing (i.e., prenatal, with the first encounter occurring during pregnancy; or postpartum, with the first encounter occurring within 365 days of delivery) and (b) diagnosis (i.e., a mood or anxiety, psychotic, substance use, or other mental health disorder, or self-harm; these categories were derived from all healthcare encounters in the perinatal period and were not mutually exclusive). In additional analyses, we required ≥2 outpatient visits (as 1 visit may be a rule-out).

Covariates were maternal age and parity, neighbourhood income quintile, rural residence, immigration status (derived from the Immigration, Refugees and Citizenship Canada Permanent Resident Database), history of recent severe violence (captured in emergency department visits in the 2 years before conception; Saunders *et al.*, 2021) and comorbidities (ascertained using Adjusted Clinical Groups in the 2 years before conception; Johns Hopkins Bloomberg School of Public Health, 2009) (Table S4).

### Statistical analyses

Baseline characteristics of women with and without TBI in the history of mental illness strata were measured using descriptive statistics and compared using standardized differences (Austin, 2009).

To address the first objective, we used modified Poisson regression (Zou, 2004) to estimate the relative risk (RR) and 95% confidence interval (CI) of perinatal mental illness, overall and by timing and type as defined above, comparing women with and without TBI. Therein, new mental illness was determined in those without a history of mental illness and recurring mental illness was estimated in those with a history of mental illness. Generalized estimating equations (Zou and Donner, 2013) were used to account for clustering due to the inclusion of more than one birth per woman in the study period. Multivariable models included age, parity, income quintile, rurality, immigration status, history of violence and comorbidities.

For the second objective, we used modified Poisson regression to examine injury-related factors associated with any new and recurring perinatal mental illness in women with TBI, using the same model covariates.

In sensitivity analyses, we tested the impacts of variations of the exposure and outcome definitions on the results; we examined TBI recorded in any diagnostic field (Hedegaard *et al.*, 2016) (sensitivity analysis 1); used the more sensitive definition of TBI (Public Health Agency of Canada, 2020) (sensitivity analysis 2) and required ≥2 outpatient visits in our definition of perinatal mental illness (sensitivity analysis 3). Finally, we stratified the analyses by maternal age, to explore whether age-related stressors could impact results (sensitivity analysis 4).

SAS version 9.4 (SAS Institute Inc., Cary, NC) was used.

## Results

Among women without a history of mental illness, there were 5,647 women with a TBI recorded in the 10 years before conception, and 507,309 without a TBI. Among those with a history of mental illness, there were 7,077 with a TBI and 279,008 without a TBI. In both cohorts, compared to women without TBI, those with TBI were more likely to be younger, primiparous, living in a rural area, non-immigrant and a victim of violence (Table 1).

**Table 1.** Baseline characteristics of women with a TBI in the 10 years before conception and those without a TBI. Reported as *n* (%) unless otherwise indicated. Cells with sample sizes <6 suppressed to minimize re-identification risk

	No history of mental illness			History of mental illness		
Baseline characteristics	TBI^a^ (*n* = 5,647)	No TBI (*n* = 507,309)	Standardized difference^b^	TBI^a^ (*n* = 7,077)	No TBI (*n* = 279,008)	Standardized difference^b^
Age (years), mean (SD)	27.53 ± 5.39	30.48 ± 4.92	0.57	27.57 ± 5.60	30.76 ± 5.24	0.59
15–19 years	373 (6.6)	9,669 (1.9)	0.23	485 (6.9)	5,910 (2.1)	0.23
20–24 years	1,340 (23.7)	47,254 (9.3)	0.40	1,786 (25.2)	28,452 (10.2)	0.40
25–29 years	1,937 (34.3)	149,506 (29.5)	0.10	2,207 (31.2)	73,825 (26.5)	0.10
30–34 years	1,391 (24.6)	198,183 (39.1)	0.31	1,778 (25.1)	103,947 (37.3)	0.26
35–39 years	525 (9.3)	87,545 (17.3)	0.24	677 (9.6)	55,294 (19.8)	0.29
40–44 years	75–79	14,330 (2.8)	0.10	136 (1.9)	10,843 (3.9)	0.12
45–49 years	<6	822 (0.2)	0.04	8 (0.1)	737 (0.3)	0.03
Multiparous	2,404 (42.6)	286,972 (56.6)	0.28	3,578 (50.6)	168,042 (60.2)	0.20
Neighbourhood income quintile (Q)						
Q1 (lowest)	1,087 (19.2)	99,363 (19.6)	0.01	1,714 (24.2)	55,992 (20.1)	0.10
Q2	1,122 (19.9)	100,684 (19.8)	0.00	1,537 (21.7)	55,288 (19.8)	0.05
Q3	1,244 (22.0)	108,677 (21.4)	0.01	1,394 (19.7)	58,100 (20.8)	0.03
Q4	1,222 (21.6)	110,038 (21.7)	0.00	1,365 (19.3)	59,694 (21.4)	0.05
Q5 (highest)	954 (16.9)	87,339 (17.2)	0.01	1,041 (14.7)	49,172 (17.6)	0.08
Missing	18 (0.3)	1,208 (0.2)	0.02	26 (0.4)	762 (0.3)	0.02
Rural residence						
Urban	4,463 (79.0)	456,131 (89.9)	0.30	5,814 (82.2)	249,007 (89.2)	0.20
Rural	1,176 (20.8)	50,663 (10.0)	0.30	1,247 (17.6)	29,637 (10.6)	0.20
Missing	8 (0.1)	515 (0.1)	0.01	16 (0.2)	364 (0.1)	0.02
Immigration status						
Long-term resident	4,889 (86.6)	334,523 (65.9)	0.50	6,562 (92.7)	231,299 (82.9)	0.30
Refugee	136 (2.4)	25,171 (5.0)	0.14	151 (2.1)	9,616 (3.4)	0.08
Family or economic class immigrant	622 (11.0)	147,615 (29.1)	0.46	364 (5.1)	38,093 (13.7)	0.29
History of violence	141 (2.5)	774 (0.2)	0.21	313 (4.4)	1,164 (0.4)	0.26
Stable chronic conditions	983 (17.4)	101,693 (20.0)	0.07	1,570 (22.2)	65,652 (23.5)	0.03
Unstable chronic conditions	630 (11.2)	54,534 (10.7)	0.01	1,083 (15.3)	37,580 (13.5)	0.05
Injury-specific variables						
≥2 TBI acute healthcare encounters	390 (0.1)	–	–	728 (0.3)	–	–
Most severe injury						
A hospitalization, discharged to facility	36 (0.0)	–	–	44 (0.0)	–	–
A hospitalization, discharged home	237 (0.0)	–	–	267 (0.1)	–	–
Emergency department visit only	5,374 (1.0)	–	–	6,766 (2.4)	–	–
≤2 years since most recent TBI acute healthcare encounter	1,372 (0.3)	–	–	1,623 (0.6)	–	–
Most recent TBI mechanism						
Motor vehicle collision	1,390 (0.3)	–	–	1,632 (0.6)	–	–
Struck by/against	2,296 (0.4)	–	–	2,985 (1.0)	–	–
Fall	1,724 (0.3)	–	–	2,087 (0.7)	–	–
Other	230–234	–	–	370–374	–	–
Missing	<6	–	–	<6	–	–
Most recent TBI intentional	260 (0.1)	–	–	571 (0.2)	–	–

a Includes individuals with a TBI recorded in ‘the most responsible diagnosis’ field in the 10 years before conception. Individuals TBI recorded outside of the ‘most responsible diagnosis’ field and those with injuries that often accompany TBI (*n* = 41,494) are excluded.

b Standardized differences >0.10 indicate clinically meaningful differences between groups.

Women with TBI were at elevated risk of both new-onset (18.5% vs. 12.7%; adjusted RR [aRR]: 1.31, 95% CI: 1.24–1.39) and recurrent mental illness perinatally (35.5% vs. 27.8%; aRR: 1.18, 95% CI: 1.14–1.22) compared to women without a TBI (Table 2). Risks were similar for both prenatal and postpartum mental illness presentations. Among women without a history of mental illness, those with TBI were at elevated risk for new-onset mood and anxiety disorders in the perinatal period; among women with a history of mental illness, those with TBI were at risk for recurrent mood and anxiety, psychotic, substance use and other mental health disorders perinatally (Table 2).

**Table 2.** Unadjusted and adjusted associations between TBI in the 10 years before conception and perinatal mental illness

	No history of mental illness (*n* = 512,956)			History of mental illness (*n* = 286,085)		
	*N* (%) with outcome	Unadjusted RR (95% CI)	Adjusted RR (95% CI)^b^	*N* (%) with outcome	Unadjusted RR (95% CI)	Adjusted RR (95% CI)^b^
**Any perinatal mental illness**						
No history of TBI	64,523 (12.7)	1.00 (Referent)	1.00 (Referent)	77,613 (27.8)	1.00 (Referent)	1.00 (Referent)
History of TBI^a^	1,047 (18.5)	1.46 (1.38–1.54)	1.31 (1.24–1.39)	2,512 (35.5)	1.27 (1.23–1.31)	1.18 (1.14–1.22)
**Prenatal mental illness**						
No history of TBI	22,825 (4.5)	1.00 (Referent)	1.00 (Referent)	30,724 (11.0)	1.00 (Referent)	1.00 (Referent)
History of TBI	350 (6.2)	1.38 (1.24–1.52)	1.31 (1.18–1.45)	983 (13.9)	1.26 (1.18–1.33)	1.20 (1.13–1.28)
**Postpartum mental illness**						
No history of TBI	41,698 (8.2)	1.00 (Referent)	1.00 (Referent)	46,889 (16.8)	1.00 (Referent)	1.00 (Referent)
History of TBI	697 (12.3)	1.50 (1.40–1.61)	1.31 (1.22–1.41)	1,529 (21.6)	1.28 (1.23–1.34)	1.17 (1.12–1.23)
**Mood or anxiety disorder**						
No history of TBI	59,012 (11.6)	1.00 (Referent)	1.00 (Referent)	72,484 (26.0)	1.00 (Referent)	1.00 (Referent)
History of TBI	976 (17.3)	1.48 (1.40–1.57)	1.34 (1.26–1.42)	2,320 (32.8)	1.25 (1.21–1.30)	1.18 (1.14–1.22)
**Psychotic disorder**						
No history of TBI	233 (0.0)	1.00 (Referent)	1.00 (Referent)	469 (0.2)	1.00 (Referent)	1.00 (Referent)
History of TBI	<6	**	**	24 (0.3)	2.01 (1.33–3.02)	1.65 (1.07–2.54)
**Substance use disorder**						
No history of TBI	1,072 (0.2)	1.00 (Referent)	1.00 (Referent)	2,349 (0.8)	1.00 (Referent)	1.00 (Referent)
History of TBI	28 (0.5)	2.33 (1.60–3.40)	1.04 (0.71–1.54)	149 (2.1)	2.49 (2.11–2.94)	1.41 (1.18–1.67)
**Another mental illness**						
No history of TBI	6,358 (1.3)	1.00 (Referent)	1.00 (Referent)	6,626 (2.4)	1.00 (Referent)	1.00 (Referent)
History of TBI	87 (1.5)	1.23 (0.99–1.52)	1.27 (1.02–1.58)	253 (3.6)	1.50 (1.32–1.70)	1.42 (1.25–1.61)
**Self-harm**						
No history of TBI	214 (0.0)	1.00 (Referent)	1.00 (Referent)	313 (0.1)	1.00 (Referent)	1.00 (Referent)
History of TBI	7 (0.1)	2.80 (1.25–6.25)	1.54 (0.72–3.29)	26 (0.4)	3.25 (2.18–4.86)	1.52 (0.99–2.33)

a Includes individuals with a TBI recorded in ‘the most responsible diagnosis’ field in the 10 years before conception. Individuals TBI recorded outside of the ‘most responsible diagnosis’ field and those with injuries that often accompany TBI (*n* = 41,494) are excluded.

b Adjusted model controls for maternal age, parity, neighbourhood income quintile, rurality, immigrant status, history of violence, and stable and unstable chronic conditions.

** Suppressed due to small sample sizes <6.

Among women with TBI without a history of mental illness, multiple TBI-related healthcare encounters were associated with an elevated risk of new-onset mental illness perinatally in unadjusted analyses. Among those with a history of mental illness, time since the most recent TBI-related healthcare encounter and intentional injuries were associated with recurrent mental illness risk (Table 3). However, with the exception of multiple TBI-related healthcare encounters, none of these variables were statistically significant after adjustment.

**Table 3.** Injury-related factors associated with perinatal mental illness among women with a TBI in the 10 years before conception^a^

	No history of mental illness (*n* = 512,956)			History of mental illness (*n* = 286,085)		
	*N* (%) with outcome	Unadjusted RR (95% CI)	Adjusted RR (95% CI)^a^	*N* (%) with outcome	Unadjusted RR (95% CI)	Adjusted RR (95% CI)^a^
Number of TBI acute healthcare encounters						
1	947 (18.0)	1.00 (Referent)	1.00 (Referent)	2,240 (35.3)	1.00 (Referent)	1.00 (Referent)
≥2	100 (25.6)	1.41 (1.17–1.71)	1.34 (1.12–1.61)	272 (37.4)	1.06 (0.96–1.18)	1.03 (0.93–1.14)
Most severe injury						
Emergency department visit only	1,010 (18.8)	1.00 (Referent)	1.00 (Referent)	2,395 (35.4)	1.00 (Referent)	1.00 (Referent)
A hospitalization	37 (13.6)	0.72 (0.53–0.97)	0.71 (0.52–0.97)	117 (37.6)	1.06 (0.91–1.24)	1.05 (0.90–1.22)
Time since most recent TBI acute healthcare encounter						
≥3 years	781 (18.3)	1.00 (Referent)	1.00 (Referent)	1,888 (34.6)	1.00 (Referent)	1.00 (Referent)
≤2 years	266 (19.4)	1.03 (0.91–1.17)	1.01 (0.89–1.15)	624 (38.4)	1.08 (1.01–1.16)	1.05 (0.98–1.13)
Most recent TBI mechanism						
Other	48 (20.5)	1.00 (Referent)	1.00 (Referent)	138 (37.2)	1.00 (Referent)	1.00 (Referent)
Struck by/against	450 (19.6)	0.94 (0.71–1.24)	0.93 (0.71–1.22)	1,038 (34.8)	0.93 (0.81–1.07)	0.93 (0.81–1.07)
Motor vehicle collision	265 (19.1)	0.92 (0.69–1.23)	0.96 (0.73–1.28)	564 (34.6)	0.93 (0.80–1.08)	0.95 (0.82–1.10)
Fall	284 (16.5)	0.80 (0.60–1.06)	0.81 (0.62–1.08)	772 (37.0)	0.99 (0.86–1.14)	1.00 (0.87–1.16)
Most recent TBI intent						
Unintentional/ undetermined/other	988 (18.4)	1.00 (Referent)	1.00 (Referent)	2,280 (35.1)	1.00 (Referent)	1.00 (Referent)
Intentional	59 (22.7)	1.24 (0.98–1.58)	1.12 (0.86–1.47)	232 (40.6)	1.16 (1.04–1.29)	1.07 (0.95–1.21)

a Adjusted model controls for maternal age, parity, neighbourhood income quintile, rurality, immigrant status, history of violence and stable and unstable chronic conditions.

Findings were similar to the main analysis when we examined TBI in any diagnostic field (sensitivity analysis 1; Table S5); used the more sensitive definition of TBI (sensitivity analysis 2; Table S6) and required ≥2 outpatient visits in our perinatal mental illness definition (sensitivity analysis 3; Table S7 and Table S8). Effect sizes were slightly larger in younger women (15–34 years) compared to older women (35–49 years) (sensitivity analysis 4; Table S9).

## Discussion

In the first study of its kind, we used whole-population data to show women with a TBI recorded in an acute healthcare encounter in the 10 years before conception were at elevated risk of both new-onset and recurrent mental illness perinatally vs. those without TBI. Findings were similar for prenatal and postpartum mental illness, with mood and anxiety disorders being the most common type of mental illness observed in both groups. In women with a TBI, having multiple acute healthcare encounters for TBI was associated with a higher risk of new mental illness perinatally. Results were robust under variations of both the TBI and outcome definitions. Our data show the need for screening for perinatal mental illness in women with TBI and for tailored prevention and treatment efforts to reduce the risk of perinatal mental illness.

There is a considerable body of literature showing the lasting impacts of TBI on mental health, mostly from professional athlete and veteran samples primarily comprised of men (Perry *et al.*, 2016). Growing evidence shows mental health is affected even more often in women with TBI than men with TBI (Farace and Alves, 2000), underscoring the importance of sex and gender-based analyses of this association. Yet, despite the importance of the perinatal period in the life course of women, including the known elevated baseline risk of mental health concerns in this period (Howard and Khalifeh, 2020), very few studies have examined the risk of perinatal mental illness associated with TBI. In their study of 208 women, Colantonio *et al.* (2010) found that depression in the postpartum period was more commonly reported in women with TBI compared to those without TBI. However, the analysis did not account for a pre-pregnancy history of mental illness, nor did it examine prenatal mental illness or diagnoses other than depression. Our study adds to the literature by showing that, in a women without active mental illness going into pregnancy, those with TBI were at elevated risk of new-onset and recurrent mental illness both pre- and postnatally, and for a range of types of mental health disorders. Our analysis of injury-related risk factors for perinatal mental illness among women with TBI is also novel, showing patients with multiple TBIs might be at greatest risk. Other injury-related variables, such as time since injury, appeared to be less important after accounting for other factors, including violence.

The impact of TBI on perinatal mental health may be indirect or direct. TBI is associated with social and health disparities that carry risks for perinatal mental illness (Chan *et al.*, 2017; Haag *et al.*, 2016). One particularly important factor is violence (Howard *et al.*, 2013). Intimate partner violence is a leading cause of TBI in women, and injuries to the head, face and neck are the most common physical injuries in women affected by intimate partner violence (Haag *et al.*, 2022; Sheridan and Nash, 2007). Indeed, in our cohort, a history of violence was much more common in women with than without TBI. The strength of the relation between TBI and perinatal mental illness was reduced after controlling for violence, and, among women with TBI, intentional injuries were associated with a particularly high risk of perinatal mental illness. Together, these data show the importance of violence in the observed associations. However, after adjustment for this and other covariates, elevated risk of perinatal mental illness remained. Some of this remaining risk may be due to a direct relation between TBI and perinatal mental illness. For example, TBI is associated with headaches, dizziness, reduced noise tolerance, irritability, sleep problems, and executive functioning and problem-solving difficulties (Kieffer-Kristensen and Teasdale, 2011; Mollayeva *et al.*, 2016; Pryor, 2004) that might be exacerbated perinatally when women have reduced opportunities for self-care and encounter additional stressors such as sleep deprivation. Perinatal hormonal fluctuations could also play a role (Brunton and Russell, 2010). Future studies should examine these potential mechanisms.

It is also important to note that the relationship between TBI and mental illness is likely bi-directional, with research showing TBI can lead to new-onset mental illness (Chan *et al.*, 2022a; Perry *et al.*, 2016; Yeh *et al.*, 2020), but may also be a consequence of pre-existing mental illness due to factors such as risk-taking behaviours and adverse life experiences (Liao *et al.*, 2012; McHugo *et al.*, 2017). While it is possible a history of mental illness could have preceded some cases of TBI in our cohort, it is notable that the relationship between TBI and perinatal mental illness persisted in women without any history of mental illness, and also increased with a rising number of prior TBI-related healthcare encounters, adding strength to the assertion that the perinatal period is a time of particular vulnerability for mental illness among women with a TBI.

### Limitations

Our findings should be interpreted in the context of a few limitations. We examined TBI in the ‘most responsible diagnosis’ field, to capture incident, rather than existing, TBI. While this approach has a positive predictive value of 97% (Hedegaard *et al.*, 2016), we may have misclassified women with polytrauma, wherein a more severe injury was listed as the most responsible diagnosis. However, findings were similar in an additional analysis in which we examined TBI in any diagnostic field. Our reliance on acute care data to capture TBI likely resulted in the inclusion of more moderate to severe injuries; we may have misclassified TBI status and underestimated the occurrence of multiple TBIs in women who did not seek healthcare for their injury or who only received outpatient care. We also may have misclassified TBI status in women whose injuries occurred more than 10 years before conception, as Ontario’s emergency department visit dataset only dates back to 2002. Further, we were unable to measure injury severity (e.g., using the Glasgow Coma Scale), an important limitation given such scores are predictive of later functional outcomes (Dos Reis Zuniga *et al.*, 2024).

Mental illness variables were captured in healthcare encounters in the publicly funded healthcare system. We may have under-ascertained perinatal mental illness for women who did not seek care for their symptoms, or who received private services (e.g., private psychologists). On the other hand, we may have over-ascertained perinatal mental illness by only requiring one outpatient visit (or one emergency department visit or hospital admission). Nevertheless, findings were similar using a more strict definition requiring two or more outpatient visits.

Finally, we had no information on race/ethnicity, gender identity, social supports and healthcare seeking behaviours. We also likely underestimated experiences of violence. These factors may have influenced our results.

### Implications

Perinatal mental illness is associated with significant adverse maternal, child and family outcomes, as well as healthcare costs (Moore *et al.*, 2021), highlighting the importance of prevention, timely identification and effective treatment. Our findings suggest women with TBI may be a high-risk group that could benefit from additional support to reduce their risk of perinatal mental illness. Such supports should start before conception and might include occupational therapy to optimize the management of TBI-related symptoms and other social risk factors, and mental health information for women planning a pregnancy. Perinatally, additional preventive measures might include in-home support from public health nurses to help women compensate for executive functioning impairment and cope with sensory overload, as well as respite services to reduce sleep deprivation in women without adequate support. To facilitate early entry into treatment, women with TBI should be screened for mental illness antenatally and early in the postpartum period. Mental health interventions should be tailored to their needs. For example, given the relationship between violence and TBI (Haag *et al.*, 2022), trauma-informed care approaches may be beneficial (Sperlich *et al.*, 2017). Given many individuals with TBI experience treatment-resistant mental illness (Naraparaeddy *et al.*, 2020), knowledge of a history of TBI should be used to inform the types of therapies and medications used.

These efforts require that providers be aware of a woman’s history of TBI. Providers should be educated about TBI with a sex and gender lens, as this has been identified as a gap in care (Hanafy *et al.*, 2022). Such training should include how to accommodate someone with a TBI and referral networks. This is necessary given reports from women of being dismissed by providers when reporting symptoms (Haag *et al.*, 2016). Toolkits such as the ‘Battered and Brain Injured’ toolkit provide education and resources on TBI and intimate partner violence (www.abitoolkit.ca).

These efforts require collaboration between rehabilitation and mental healthcare providers, suggesting the benefits of integrated care approaches that promote coordinated, patient-centred care (Chan *et al.*, 2022b). This is particularly important as both TBI and mental health diagnoses can serve as exclusion criteria for many services.

## Conclusion

In a large, population-based study, history of TBI was associated with increased risk of new-onset and recurrent mental illness in the perinatal period. These findings demonstrate the need for providers to be attentive to the risk for perinatal mental illness in women with a TBI. This population may benefit from mental illness preventive efforts, screening and tailored treatment options.

## Supporting information

Brown et al. supplementary material Brown et al. supplementary material

## Data Availability

Data used for this study were housed at ICES, an independent not-for-profit corporation. The dataset used in this study is held securely in coded form at ICES. While the data sharing agreements that govern the dataset prohibit making it publicly available, access may be granted to those who meet specified criteria for confidential access. External individuals must apply for access through ICES’ Data and Analytic Services (DAS), a division of ICES established to provide data and analytic services to ‘third party researchers’. The dataset that approved third party researchers would be permitted to access would be adjusted to ensure the risk of re-identification of any underlying individuals is low. Information about the application process, including the DAS Data Request Form and the criteria for access, including, for example, confirmation of approval by a Research Ethics Board, are available at https://www.ices.on.ca/DAS/Submitting-your-request. For general information visit www.ices.on.ca/DAS or email das@ices.on.ca.

## Supplementary material

The supplementary material for this article can be found at https://doi.org/10.1017/S2045796025000150.
