# Supplementary material for: A population-based cohort study of perinatal mental illness following traumatic brain injury
Source: Epidemiol Psychiatr Sci. 2025 Mar 13;34:e19. doi: 10.1017/S2045796025000150 (PMC11955423; doi:10.1017/S2045796025000150)
Supplement: Brown et al. supplementary material [file S2045796025000150sup001.docx]

**Table S1. Description of the ICES datasets.**

| **Data source** | **Variable** | **Coding structure** | **Inception** |
| --- | --- | --- | --- |
| Canadian Institute for Health Information Discharge Abstract Database | Hospital admissions | Canadian Coding Standards for the International Classification of Diseases and Related Health Problems codes for diagnoses and Canadian Classification of Health Interventions codes for procedures | 1988 |
| Census | Sociodemographic data | N/A | 2006, 2011, 2016 |
| Immigrants, Refugees, and Citizenship Canada Permanent Residents Database | Immigration status | N/A | 1985 |
| National Ambulatory Care Reporting System | Emergency department visits | Canadian Coding Standards for the International Classification of Diseases and Related Health Problems codes for diagnoses and Canadian Classification of Health Interventions codes for procedures | 2000 |
| Ontario Health Insurance Database | Outpatient physician visits | Physician billing codes | 1991 |
| Ontario Mental Health Reporting System | Psychiatric hospital admissions | Diagnostic and Statistical Manual of Mental Disorders | 2005 |
| Registered Persons Database | Sociodemographic data | N/A | 1991 |

**Figure S1. Study flowchart.**

**
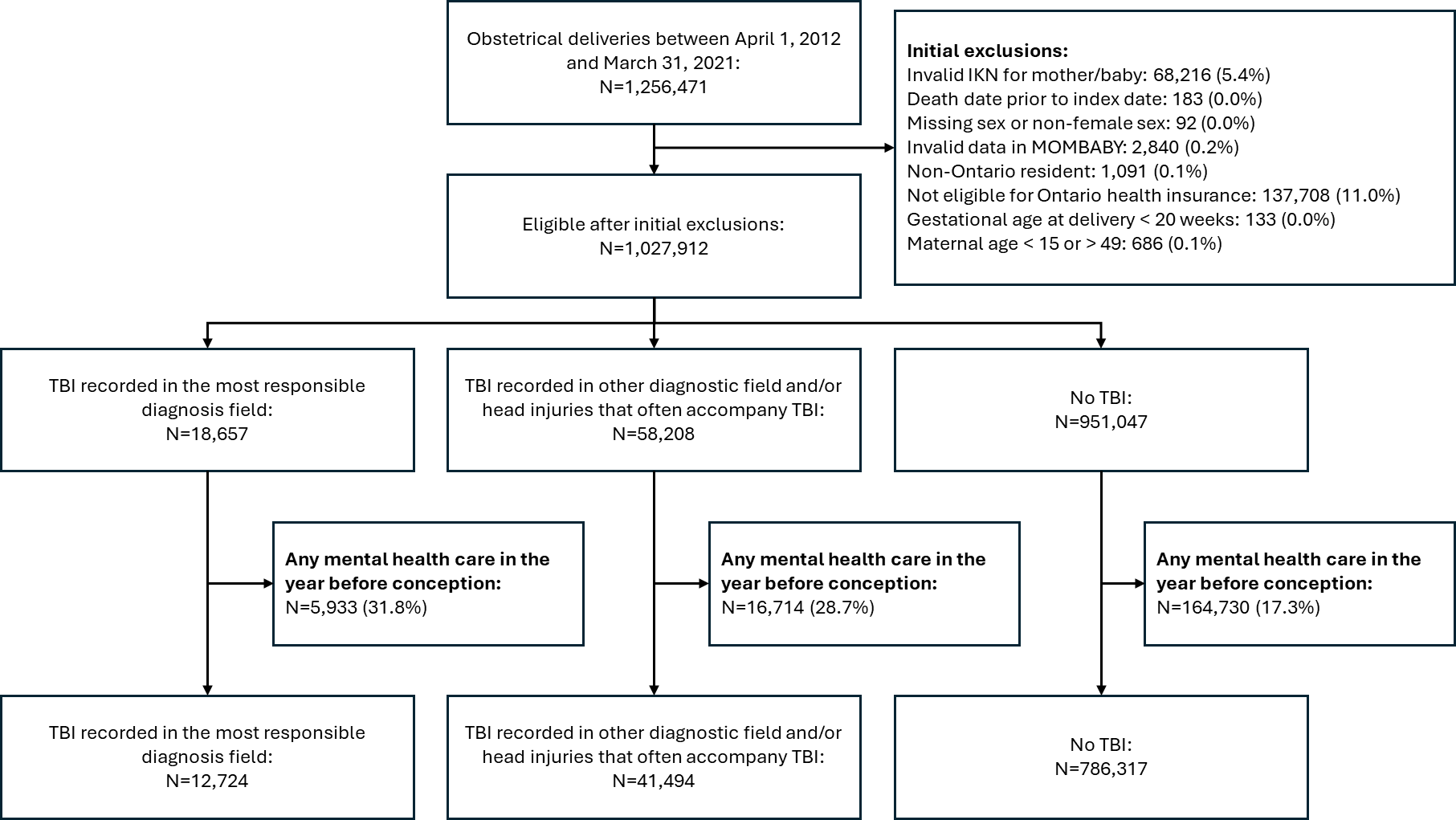
**

**Table S2. Ascertainment of study exposures.**

| **Variable** | **Definition** | **Data sources** |
| --- | --- | --- |
| Traumatic brain injury (main definition) | ≥ 1 emergency department visits or hospitalizations associated with any of the following codes in the most responsible diagnosis field in the 10 years prior to conception: S02.0, S02.1, S02.8, S02.9, S04.0, S06.0-S06.9, S07.1 | CIHI-DAD, NACRS |
| Traumatic brain injury (in any diagnostic field) | ≥ 1 emergency department visits or hospitalizations associated with any of the following codes in any diagnosis field in the 10 years prior to conception: S02.0, S02.1, S02.8, S02.9, S04.0, S06.0-S06.9, S07.1 | CIHI-DAD, NACRS |
| Traumatic brain injury (sensitive definition) | ≥ 1 emergency department visits or hospitalizations associated with any of the following codes in the most responsible diagnosis field in the 10 years prior to conception: S01.0-S01.9, S02.0, S02.1, S02.3, S02.7-S02.9, S04.0, S06.0-S06.9, S07.0, S07.1, S07.8, S07.9, S09.7-S09.9, T90.1, T90.2, T90.4, T90.5, T90.8, or T90.9 | CIHI-DAD, NACRS |
| Number of acute healthcare encounters for TBI | Number of health care encounters for a TBI in the 10 years before conception separated by at least 24 hours | CIHI-DAD, NACRS |
| Severity of the most severe TBI | Injury severity (using hospital admission and discharge destination as proxies), classified as (1) hospitalized and discharged to another facility: discharge disposition 01, 10 / Transfer to long-term care or ambulatory/palliative care: discharge disposition 02, 03, 20, 30, 40; (2) hospitalized and went home: Discharged home: discharge disposition 04, 05, Signing out against medical advice, not returning from a pass, etc.: discharge disposition 06, 61, 62, 65; or (3) no hospital admissions – TBI was only recorded in NACRS | CIHI-DAD, NACRS |
| Time since the most recent acute healthcare encounter for TBI | Time since most recent health care contact, measured in years between the most recently recorded TBI encounter and conception | CIHI-DAD, NACRS |
| Mechanism of injury | Mechanism of injury for the most recent incident TBI health care encounter, classified as (1) struck by/against (X79, Y00, Y04, Y29, Y35.3, W20–W22,W50–W52); (2) motor vehicle collision (X82, Y03, Y32, Y36.1, V01–V99); (3) falls (X80, Y01, Y30, W00–W19); or (4) other (U01, U02, U03.0, W23–W45, W49, W53–W84, W85–W99, X00–X78, X83–X99, Y02, Y05–Y28, Y31, Y33, Y34, Y35 (except .3), Y36 (except .1), Y85–Y87, Y89) | CIHI-DAD, NACRS |
| Intent | Intent for the most recent TBI health care encounter, classified as: (1) assault / self-harm (U01, U02, U03.0, U03.9, X60–X92, X93–X99, Y00–Y09, Y87.0, Y87.1); or (2) unintentional / undetermined (V01–V99, W00–W45, W49–W99, X00–X19, X20–X39, X40–X58, Y10–Y19, Y20, Y21–Y36, Y85, Y86, Y87.2, Y89) | CIHI-DAD, NACRS |

Abbreviations: CIHI-DAD = Canadian Institute for Health Information Discharge Abstract Database; NACRS = National Ambulatory Care Reporting System.

**Table S3. Ascertainment of study outcomes.**

| **Variable** | **Definition** | **Data sources** |
| --- | --- | --- |
| Mood or anxiety disorder | a. Psychiatrist [SPEC=19] and outpatient (LOCATION: O, L, H, P) and non-lab service [substr(FEECODE,1,1) ne 'G'] OR Family physician / general practitioner / pediatrician [SPEC=00, SPEC=26] and mental illness or addiction diagnosis code [DXCODE] and outpatient (LOCATION: O, L, H, P) and non-lab service [substr(FEECODE,1,1) ne 'G'] and MHA diagnostic code [DXCODE] and fee code (FEECODE=K122 or K123 or K704), where DXCODES = 296, 300, 309, 311  b. CIHI-DAD/NACRS: Before 2016/17: DX10CODE1 F30-F34, F38-F43, F48.8, F48.9, F53.0, F93.1-F93.2; 2016/17-present: DX10CODE1 F06.3, F06.4, F30-F34, F38-F43, F45.2, F53.0, F63.3, F93.0-F93.2, F94.0-F94.2  c. OMHRS: Before 2016/17: AXIS1_DSM4CODE_DISCH1 = 296.x (all 296 codes), 300, 300.0x, 300.2x, 300.3x, 300.4x, 301.13, 308.3x, 309.0x, 309.24, 309.28, 309.3x, 309.4x, 309.8x, 309.9x, 311; Provisional: 6, 7, 15. 2016/17-2018/19: DSM5CODE_DISCH1 = 293.83, 293.84, 296.x (all 296 codes), 300, 300.0x, 300.2x, 300.3x, 300.4x, 300.7x, 301.13, 308.3x, 309, 309.0x, 309.21, 309.24, 309.28, 309.3x, 309.4x, 309.81, 309.89, 309.9x, 311.x, 312.39, 313.23, 313.89, 625.4, 698.4x, Provisional = 3-7; 2019/20 to present: ICD10CMCODE_DISCH1=F06.3, F06.4, F06.8, F31-F34, F40.0-F40.2, F41.0, F41.1, F41.8, F41.9, F42.2-F42.4, F42.8, F42.9, F43.0-F43.2, F43.8, F43.9, F45.2, F63.3, F91.4, F94.2, F93.0, F94.0-F94.2; Provisional = 3-7 | CIHI-DAD,  NACRS,  OHIP, OMHRS |
| Psychotic disorder | a. Psychiatrist [SPEC=19] and outpatient (LOCATION: O, L, H, P) and non-lab service [substr(FEECODE,1,1) ne 'G'] OR Family physician / general practitioner / pediatrician [SPEC=00, SPEC=26] and mental illness or addiction diagnosis code [DXCODE] and outpatient (LOCATION: O, L, H, P) and non-lab service [substr(FEECODE,1,1) ne 'G'] and MHA diagnostic code [DXCODE] and fee code (FEECODE=K122 or K123 or K704), where DXCODES = 295, 297, 298  b. CIHI-DAD/NACRS: DX10CODE1 F20 (excluding F20.4), F22-F25, F28-F29, F53.1  c. OMHRS: Before 2016/17: AXIS1_DSM4CODE_DISCH1 = 295.x (all 295 codes), 297.x (all 297 codes), 298.x (all 298 codes); Provisional = 5; 2016/17-2018/19: DSM5CODE_DISCH1 = 293.81, 293.82, 295.x (all 295 codes), 297.x (all 297 codes), 298.x (all 298 codes), Provisional = 2; 2019/20 to present: ICD10CMCODE_DISCH1=F20.81, F20.9, F22, F23, F25, F06.0-F06.2, F28, F29; Provisional = 2 | CIHI-DAD,  NACRS,  OHIP, OMHRS |
| Substance use disorder | a. Psychiatrist [SPEC=19] and outpatient (LOCATION: O, L, H, P) and non-lab service [substr(FEECODE,1,1) ne 'G'] OR Family physician / general practitioner / pediatrician [SPEC=00, SPEC=26] and mental illness or addiction diagnosis code [DXCODE] and outpatient (LOCATION: O, L, H, P) and non-lab service [substr(FEECODE,1,1) ne 'G'] and MHA diagnostic code [DXCODE] and fee code (FEECODE=K122 or K123 or K704), where DXCODES = 291, 292, 303, 304  b. CIHI-DAD/NACRS: DX10CODE1 F10-F19, F55  c. OMHRS: Before 2016/17: AXIS1_DSM4CODE_DISCH1 = 291.x (all 291 codes, excluding 291.82), 292.x (all 292 codes, excluding 292.85), 303.x (all 303 codes), 304.x (all 304 codes), 305.x (all 305 codes), PROVDX_DSM4CODE_ADM1: 4; 2016/17-2018/19: DSM5CODE_DISCH1 = 291.x (all 291 codes), 292.x (all 292 codes), 303.x (all 303 codes), 304.x (all 304 codes), 305.x, Provisional = 16; 2019/20 to present: ICD10CMCODE_DISCH1=F10-F19, Z72.0; Provisional = 16 | CIHI-DAD,  NACRS,  OHIP, OMHRS |
| Other mental illness | a. Psychiatrist [SPEC=19] and outpatient (LOCATION: O, L, H, P) and non-lab service [substr(FEECODE,1,1) ne 'G'] OR Family physician / general practitioner / pediatrician [SPEC=00, SPEC=26] and mental illness or addiction diagnosis code [DXCODE] and outpatient (LOCATION: O, L, H, P) and non-lab service [substr(FEECODE,1,1) ne 'G'] and MHA diagnostic code [DXCODE] and fee code (FEECODE=K122 or K123 or K704), where DXCODES = 301, 302, 306, 307, 309, 313-315  b. CIHI-DAD/NACRS: DX10CODE1 All other F06-F99 from above not included in other categories, and excluding IDD diagnoses  c. OMHRS: All other OMHRS from above not included in other categories, and excluding IDD diagnoses | CIHI-DAD,  NACRS,  OHIP, OMHRS |
| Self-harm | ICD-10: DX10CODE2-10 X60-X84, Y10-Y19, Y28; MANNER_OF_DEATH in ORGD (excluding self-harm attached to a new TBI diagnosis) | NACRS, ORGD |

Abbreviations: CIHI-DAD = Canadian Institute for Health Information Discharge Abstract Database; NACRS = National Ambulatory Care Reporting System; OHIP = Ontario Health Insurance Plan dataset; OMHRS = Ontario Mental Health Reporting System.

**Table S4. Ascertainment of study covariates.**

| **Variable** | **Definition** | **Data sources** |
| --- | --- | --- |
| Age | Defined on conception date, in years | MOMBABY |
| Parity | Defined on conception date, and categorized as primiparous (no previous deliveries) or multiparous (1 or more previous deliveries) | MOMBABY |
| Neighbourhood income quintile | Dissemination area-level median household income, divided into quintiles | RPDB, Census |
| Rural residence | Living in a town or municipality outside the commuting zone of a larger urban centre with population of 10,000 or more | RPDB, Census |
| Immigration status | Classified as refugee, family or economic class immigrant, or long-term immigrant | IRCC |
| Severe violence | ≥ 1 hospitalizations or emergency department visits (without admission) in the 2 years prior to conception for external causes of morbidity and mortality related to assault: ICD-10: X85 to Y09 | CIHI-DAD, NACRS |
| Comorbidities | Ascertained in the 2 years prior to conception using the Johns Hopkins Adjusted Clinical Groups and classified as stable or unstable based on healthcare use patterns | CIHI-DAD, NACRS, OHIP |

Abbreviations: CIHI-DAD = Canadian Institute for Health Information Discharge Abstract Database; IRCC = Immigration, Refugees and Citizenship Canada database; NACRS = National Ambulatory Care Reporting System; OHIP = Ontario Health Insurance Plan dataset; RPDB = Registered Persons Database.

**Table S5. Sensitivity analysis 1: Unadjusted and adjusted associations between TBI in the 10 years before conception and perinatal mental illness, examining TBI in any diagnostic field.**

|  | **No history of mental illness (n=513,533)** | | | **History of mental illness (n=286,894)** | | |
| --- | --- | --- | --- | --- | --- | --- |
|  | **N (%) with outcome** | **Unadjusted**  **RR (95% CI)** | **Adjusted RR**  **(95% CI)^b^** | **N (%) with outcome** | **Unadjusted**  **RR (95% CI)** | **Adjusted RR**  **(95% CI)^b^** |
| **Any perinatal mental illness** |  |  |  |  |  |  |
| No history of TBI | 64,523 (12.7) | 1.00 (Referent) | 1.00 (Referent) | 77,613 (27.8) | 1.00 (Referent) | 1.00 (Referent) |
| History of TBI^a^ | 1,154 (18.5) | 1.46 (1.38-1.54) | 1.31 (1.24-1.39) | 2,802 (35.5) | 1.27 (1.23-1.31) | 1.19 (1.15-1.22) |

^a^ Includes individuals with a TBI recorded in any diagnostic field using the specific definition of TBI. Individuals with injuries that often accompany TBI (n=40,108) are excluded.

^b^ Adjusted model controls for maternal age, parity, neighbourhood income quintile, rurality, immigrant status, history of violence, and stable and unstable chronic conditions.

**Table S6. Sensitivity analysis 2: Unadjusted and adjusted associations between TBI in the 10 years before conception and perinatal mental illness, using the sensitivity definition of TBI.**

|  | **No history of mental illness (n=530,889)** | | | **History of mental illness (n=305284)** | | |
| --- | --- | --- | --- | --- | --- | --- |
|  | **N (%) with outcome** | **Unadjusted**  **RR (95% CI)** | **Adjusted RR**  **(95% CI)^b^** | **N (%) with outcome** | **Unadjusted**  **RR (95% CI)** | **Adjusted RR**  **(95% CI)^b^** |
| **Any perinatal mental illness** |  |  |  |  |  |  |
| No history of TBI | 64,523 (12.7) | 1.00 (Referent) | 1.00 (Referent) | 77,613 (27.8) | 1.00 (Referent) | 1.00 (Referent) |
| History of TBI^a^ | 3,942 (16.7) | 1.31 (1.27-1.35) | 1.20 (1.16-1.23) | 8,887 (33.8) | 1.21 (1.19-1.23) | 1.14 (1.12-1.16) |

^a^ Includes individuals with a TBI recorded in “the most responsible diagnosis” field in the 10 years before conception using the sensitive definition of TBI. Individuals with TBI recorded outside of the “most responsible diagnosis” field (n=4,362) are excluded.

^b^ Adjusted model controls for maternal age, parity, neighbourhood income quintile, rurality, immigrant status, history of violence, and stable and unstable chronic conditions.

**Table S7. Sensitivity analysis 3: Unadjusted and adjusted associations between TBI in the 10 years before conception and any perinatal mental illness, requiring ≥ 2 outpatient visits (vs. 1 visit) in the definition of perinatal mental illness.**

|  | **No history of mental illness (n=512,956)** | | | **History of mental illness (n=286,085)** | | |
| --- | --- | --- | --- | --- | --- | --- |
|  | **N (%) with outcome** | **Unadjusted**  **RR (95% CI)** | **Adjusted RR**  **(95% CI)^b^** | **N (%) with outcome** | **Unadjusted**  **RR (95% CI)** | **Adjusted RR**  **(95% CI)^b^** |
| **Any perinatal mental illness** |  |  |  |  |  |  |
| No history of TBI | 25,837 (5.1) | 1.00 (Referent) | 1.00 (Referent) | 40,608 (14.6) | 1.00 (Referent) | 1.00 (Referent) |
| History of TBI^a^ | 513 (9.1) | 1.78 (1.64-1.94) | 1.49 (1.37-1.62) | 1,481 (20.9) | 1.43 (1.36-1.50) | 1.28 (1.22-1.34) |

^a^ Includes individuals with a TBI recorded in “the most responsible diagnosis” field in the 10 years before conception. Individuals with TBI recorded outside of the “most responsible diagnosis” field and those with injuries that often accompany TBI (n=41,494) are excluded.

^b^ Adjusted model controls for maternal age, parity, neighbourhood income quintile, rurality, immigrant status, history of violence, and stable and unstable chronic conditions.

^c^ Interaction between TBI and remote history of mental illness.

**Table S8. Sensitivity analysis 3: Injury-related factors associated with perinatal mental illness, requiring ≥ 2 outpatient visits (vs. 1 visit) in the definition of perinatal mental illness, among women with a TBI in the 10 years before conception.^a^**

|  | **No history of mental illness (n=512,956)** | | | **History of mental illness (n=286,085)** | | |
| --- | --- | --- | --- | --- | --- | --- |
|  | **N (%) with outcome** | **Unadjusted**  **RR (95% CI)** | **Adjusted RR**  **(95% CI)^b^** | **N (%) with outcome** | **Unadjusted**  **RR (95% CI)** | **Adjusted RR**  **(95% CI)^b^** |
| Number of TBI encounters |  |  |  |  |  |  |
| 1 | 458 (8.7) | 1.00 (Referent) | 1.00 (Referent) | 1,310 (20.6) | 1.00 (Referent) | 1.00 (Referent) |
| ≥ 2 | 55 (14.1) | 1.61 (1.23-2.11) | 1.49 (1.14-1.95) | 171 (23.5) | 1.14 (0.99-1.32) | 1.10 (0.95-1.26) |
| Most severe injury |  |  |  |  |  |  |
| No hospitalization | 493 (9.2) | 1.00 (Referent) | 1.00 (Referent) | 1,413 (20.9) | 1.00 (Referent) | 1.00 (Referent) |
| A hospitalization | 20 (7.3) | 0.80 (0.52-1.23) | 0.78 (0.50-1.21) | 68 (21.9) | 1.04 (0.83-1.30) | 1.02 (0.82-1.28) |
| Time since most recent TBI encounter |  |  |  |  |  |  |
| ≥ 3 years | 366 (8.6) | 1.00 (Referent) | 1.00 (Referent) | 1,078 (19.8) | 1.00 (Referent) | 1.00 (Referent) |
| **≤** 2 years | 147 (10.7) | 1.22 (1.02-1.47) | 1.17 (0.97-1.41) | 403 (24.8) | 1.24 (1.12-1.37) | 1.16 (1.04-1.28) |
| Most recent TBI mechanism |  |  |  |  |  |  |
| Other | 22 (9.4) | 1.00 (Referent) | 1.00 (Referent) | 84 (22.6) | 1.00 (Referent) | 1.00 (Referent) |
| Motor vehicle collision | 226 (9.8) | 1.04 (0.68-1.61) | 1.03 (0.67-1.58) | 634 (21.2) | 0.93 (0.76-1.14) | 0.93 (0.76-1.13) |
| Struck by/against | 129 (9.3) | 0.99 (0.64-1.56) | 1.05 (0.67-1.63) | 318 (19.5) | 0.85 (0.69-1.05) | 0.90 (0.73-1.12) |
| Fall | 136 (7.9) | 0.84 (0.54-1.32) | 0.87 (0.56-1.36) | 445 (21.3) | 0.94 (0.76-1.15) | 0.98 (0.80-1.20) |
| Most recent TBI intent |  |  |  |  |  |  |
| Unintentional/undetermined/other | 475 (8.8) | 1.00 (Referent) | 1.00 (Referent) | 1,322 (20.3) | 1.00 (Referent) | 1.00 (Referent) |
| Violence/self-harm | 38 (14.6) | 1.64 (1.21-2.24) | 1.45 (1.02-2.06) | 159 (27.8) | 1.38 (1.19-1.59) | 1.14 (0.96-1.34) |

^a^ Includes individuals with a TBI recorded in “the most responsible diagnosis” field in the 10 years before conception. Individuals with TBI recorded outside of the “most responsible diagnosis” field and those with injuries that often accompany TBI (n=41,494) are excluded.

^b^ Adjusted model controls for maternal age, parity, neighbourhood income quintile, rurality, immigrant status, history of violence, and stable and unstable chronic conditions.

**Table S9. Sensitivity analysis 4: Unadjusted and adjusted associations between TBI in the 10 years before conception and perinatal mental illness, stratified by maternal age.**

|  | **No history of mental illness (n=512,956)** | | | **History of mental illness (n=286,085)** | | |
| --- | --- | --- | --- | --- | --- | --- |
|  | **N (%) with outcome** | **Unadjusted**  **RR (95% CI)** | **Adjusted RR**  **(95% CI)^a^** | **N (%) with outcome** | **Unadjusted**  **RR (95% CI)** | **Adjusted RR**  **(95% CI)^a^** |
| **15 to 34 year-olds** |  |  |  |  |  |  |
| No history of TBI | 52,569 (13.0) | 1.00 (Referent) | 1.00 (Referent) | 59,973 (28.3) | 1.00 (Referent) | 1.00 (Referent) |
| History of TBI | 966 (19.2) | 1.47 (1.39-1.56) | 1.38 (1.30-1.46) | 2,246 (35.9) | 1.26 (1.22-1.31) | 1.21 (1.17-1.26) |
|  |  |  |  |  |  |  |
| **35-49 year-olds** |  |  |  |  |  |  |
| No history of TBI | 11,954 (11.6) | 1.00 (Referent) | 1.00 (Referent) | 17,640 (26.4) | 1.00 (Referent) | 1.00 (Referent) |
| History of TBI | 81 (13.4) | 1.15 (0.94-1.41) | 1.11 (0.90-1.36) | 266 (32.4) | 1.22 (1.11-1.35) | 1.19 (1.08-1.32) |

^a^ Adjusted model controls for parity, neighbourhood income quintile, rurality, immigrant status, history of violence, and stable and unstable chronic conditions.
